# Biopolymeric Films of Amphiphilic Derivatives of Chitosan: A Physicochemical Characterization and Antifungal Study

**DOI:** 10.3390/ijms20174173

**Published:** 2019-08-26

**Authors:** Anna Carolina Rodrigues Santos Alves, Aline Margarete Furuyama Lima, Marcio José Tiera, Vera Aparecida de Oliveira Tiera

**Affiliations:** Department of Chemistry and Environmental Sciences, Institute of Biosciences, Humanities and Exact Sciences, São Paulo State University, São José do Rio Preto, SP 15054-000, Brazil

**Keywords:** chitosan, derivatives, films, antifungal activity, coating, edible films

## Abstract

The chemical modification of chitosan has been an active subject of research in order to improve the physicochemical and antifungal properties of chitosan-based films. The aim of this study was to evaluate the physiochemical and antifungal properties of films prepared with chitosan and its derivatives containing diethylaminoethyl (DEAE) and dodecyl groups (Dod). Chitosans and selected derivatives were synthesized and characterized, and their films blended with glycerol and sorbitol (5%, 10%, and 20%). They were studied by means of the evaluation of their mechanical, thermal, barrier, and antifungal properties. The collected data showed that molecular weight (Mw), degree of acetylation, and grafting with DEAE and Dod groups greatly affected the mechanical, thickness, color, and barrier properties, all of which could be tailored by the plasticizer percentage. The antifungal study against *Aspergillus flavus*, *Alternaria alternata*, *Alternaria solani*, and *Penicillium expansum* showed that the films containing DEAE and Dod groups exhibited higher antifungal activity than the non-modified chitosans. The mechanical properties of highly soluble films were improved by the plasticizers at percentages of 5% and 10%, indicating these derivatives as potential candidates for the coating of seeds, nuts and fruits of various crops.

## 1. Introduction

Food products are subject to fungal and bacterial contamination, causing undesirable reactions that impair taste, odor, color, and texture properties, resulting in products of low sensory quality that are inappropriate for consumption. In packaged foods, for example, contamination has a significant impact on quality, which depends on a variety of intrinsic and extrinsic factors [1]. To enhance the safety and shelf life of ready-to-eat foods, synthetic additives have been widely used. However, they have gradually lost market share due to health barriers and government concerns over the consequences for public health. In this regard, natural or semisynthetic additives for food preservation have been developed to prevent the growth of pathogens, assuring a differential in quality and supporting market expansion [2,3]. In particular, chitosan has been considered a potential preservative for food products due to its antimicrobial activity and other remarkable properties such as biodegradability and its nontoxic character. Chitosan also has the ability to form films that may be used to improve food quality and shelf life [4,5]. For example, it can be used as an edible film for the protection of seeds [6] and fresh food products [7], and its carboxymethyl derivative marketed as Nutrasave^®^ was recently launched as an innovative coating for fresh fruits [8].

The antimicrobial activity of chitosan has been reported against different microorganisms such as bacteria, fungi, and yeasts [9]. Although chitosan has favorable properties to be employed in the food protection field, one of its limitations is the low solubility at neutral pH due to the pKa (6.2–6.4) of its amino groups. Moreover, films prepared only from chitosan solution usually do not have appropriate properties; therefore, during the process and depending on the application intended, either plasticizers or crosslinking agents may be required to improve their mechanical and film forming properties [10,11]. To overcome these limitations, the chemical modification of chitosan is an alternative that may provide the needed properties while increasing the antifungal action and mechanical properties of this polysaccharide. The grafting of varied groups onto the chitosan backbone can modify its physicochemical properties, increasing the solubility and the antimicrobial activity against plant and animal pathogenic fungi and bacteria [12]. In this respect, chitosan, having a high degree of deacetylation and modified with succinic anhydride, showed increased activity against the gram-positive bacterium *Staphylococcus aureus* and the gram-negative bacterium *Escherichia coli* [13]. A similar approach was employed by Xing et al. [14] with an oleoyl-modified chitosan derivative, which demonstrated increased inhibitory indexes against the fungi *Nigrospora sphaerica*, *Botryosphaeria dothidea*, *Nigrospora oryzae*, *Alternaria tenuissima*, *Gibberella seae*, and *Fusarium culmorum*. Other approaches involving the addition of small molecules [15], metal ions [16], clays [17], oils [18], and other additives [19] to formulations are also frequently used to improve the mechanical and antimicrobial activity of films. 

Previous studies carried out by our research group showed that amphiphilic derivatives of chitosan exhibited excellent inhibition activity against the fungi *Aspergillus flavus* and *Aspergillus parasiticus*. The derivatives substituted with varied contents of dodecyl groups and hydrophilic groups such as propyltrimethylammonium [20], pentyltrimethylammonium [21], and diethylaminoethyl [22] displayed high inhibition indexes against these fungi. In addition, the antifungal activity of these amphiphilics has been shown to be strongly dependent on the molecular mass, the hydrophobic content, and the concentration of the polymers. In the present work, it was hypothesized that low molecular weight amphiphilic derivatives of chitosan could be used to form films with improved antimicrobial activity against some common fungi. A derivative with low molecular weight (Mw), and containing diethylaminoethyl (DEAE) groups was selected because of its high activity against the fungi of the genus *Aspergillus* [22,23]. A comparative study was performed using chitosan of varied Mw for the preparation of films with the aims of investigating the film properties and their antifungal action against the fungi *Aspergillus flavus*, *Alternaria solani*, *Alternaria alternate*, and *Penicillium expansum*. 

## 2. Results and Discussion

### 2.1. Synthesis and Characterization of the Chitosan Derivatives

**Scheme 1:**
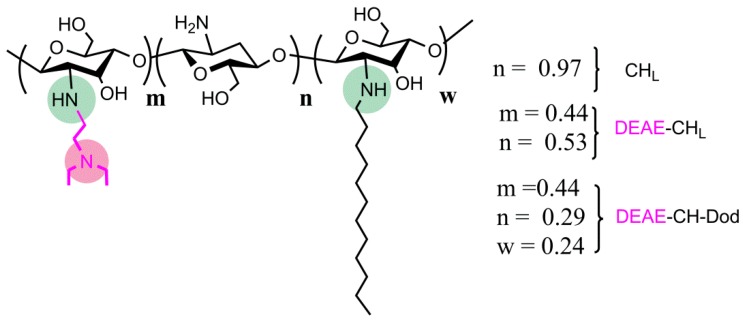
Schematic representation of low molecular weight chitosan (CH_L_), diethylaminoethyl-chitosan (DEAE-CH_L_) and its dodecylated derivative (DEAE-CH_L_-Dod)

Aiming to comparatively evaluate the effects of Mw and the chemical grafting on the physicochemical and antimicrobial properties of the chitosan films, five samples with varied Mw and degree of deacetylation (DDA) were selected for the study (Table 1): a commercial sample with DDA of 77% (CH_C_), a highly deacetylated sample (CH_H_, DDA 97%), its low-Mw sample (CH_L_), and its amphiphilic derivatives containing diethylaminoethyl (DEAE-CH_L_) and dodecyl (DEAE-CH_L_-Dod) groups (Scheme 1). All chitosan samples were characterized as previously described by ^1^H-NMR and GPC measurements (Figures S1 and S2). As expected, the deacetylation process performed in aqueous NaOH solution increased the polydispersity (PDi) of CH_H_ compared to the starting chitosan CH_c_. On the other hand, the degradation process, followed by the purification process by extensive dialysis, decreased PDi values to about 2.5 (Table 1). These samples were used to prepare films without and with 5%, 10%, and 20% of the plasticizers glycerol and sorbitol and their physicochemical and antimicrobial activities were evaluated comparatively. 

### 2.2. Films X-Ray Diffraction Study

**Figure 1:**
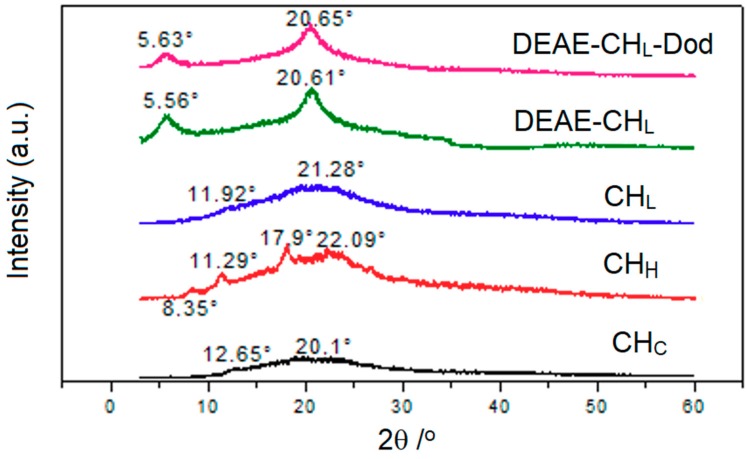
X-ray diffraction for films of commercial chitosan and its derivatives.

X-ray diffractograms of all films without plasticizers are shown in Figure 1. In general, films prepared by casting without plasticizers displayed amorphous structures denoted by the broad peak centered at 2θ~20°. The CH_C_ diffractogram shows only a broad peak centered at 2θ~20°, while deacetylated chitosan (CH_H_) film displayed three peaks at 2θ = 8.35°, 11.29°, and 17.90° which have been attributed to the hydrated crystalline region (8.35°, 11.29°) and the amorphous region (17.90°) [24]. In contrast to CH_L_, the diffractograms of films prepared with its derivatives DEAE-CH_L_ and DEAE-CH_L_-Dod are distinct by the appearance of a peak centered at 2θ~5.6°, which indicates the formation of crystalline regions resulting from the grafting with DEAE groups. Although the DEAE group is a tertiary amino group, it gives the polymer chain an amphipathic character due its methyl and methylene groups. Similar appearances have been observed for *N*-octanoyl and *N*-myristoyl chitosans [25], suggesting that DEAE-CH_L_ has a more crystalline organization than that obtained for the film with CH_L_.

The addition of plasticizers did not significantly affect the diffractograms of the films and, for all formulations, a similar profile was observed. In general, for films prepared with CH_C_, the addition of glycerol (Gly) and sorbitol (Sor) intensified the peaks at 2θ = 8.35°, 11.29° and 17.90°, which were barely seen in the absence of plasticizer, suggesting an increase in a hydrated crystal pattern [26]. This result indicated that a small amount of plasticizer could promote the crystallization of chitosan (Figure S3). Similarly, the addition of Gly and Sor to deacetylated chitosan CH_H_ and CH_L_ films did not significantly affect their structural ordering. The films obtained with DEAE-CH_L_ and DEAE-CH_L_-Dod derivatives in the presence of 20% glycerol became less crystalline, indicated by the decreased intensity for the peak at 2θ ≈ 5.6° (Figure S4). 

### 2.3. Thermal Analyses

**Figure 2:**
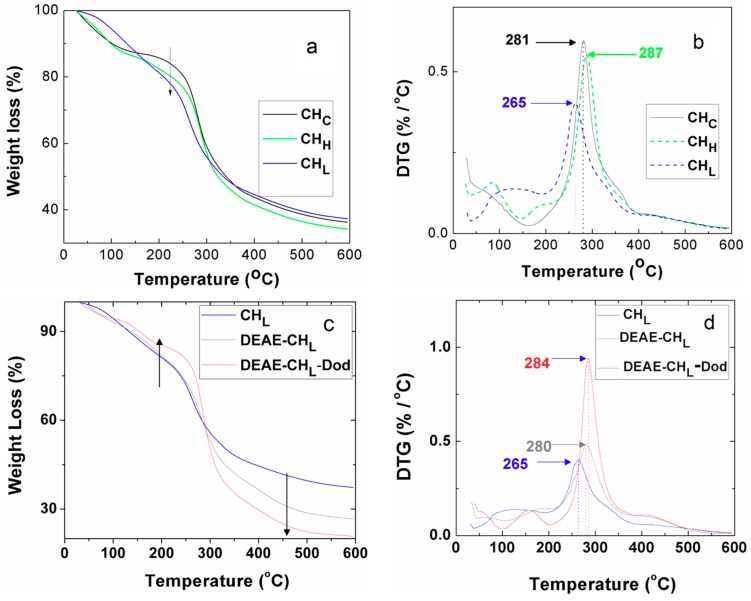
TGA and DTGA curves for films of (**a**,**b**) commercial (CH_C_) and deacetylated chitosans (CH_H_ and CH_L_) and (**c**,**d**) the amphiphilic derivatives (DEAE-CH_L_ and DEAE-CH_L_-Dod) of chitosan. (Upward arrow indicates increasing stability; Downward arrow indicates decreasing residual mass).

Figure 2 shows the thermogravimetric (TG) and its derivative (DTG) analyses for the films of chitosan and its derivatives. The thermograms are characterized by a two-step weight loss, the first being related to the loss of water. As can be seen in Figure 2a, the first thermal event occurs from the start of the run up to 200 °C and refers to the loss of water adsorbed on the chitosan chain and bound via hydrogen bonding, whereas the second thermal event occurs in the range from 250 °C to 450 °C and refers to the thermal degradation of the chitosan chain [27,28]. In this range, the major loss of weight corresponding to degradation of the chitosan chain occurred. From Figure 2b, it is also seen that the temperature for the maximum degradation rate for CH_C_, CH_H_, and CH_L_ is first displaced to a higher temperature (from 281 °C to 287 °C) when the degree of acetylation (DA) was increased from 77% to 97%, and decreased from 287 °C to 265 °C when Mw was decreased from 143 kDa to 11 kDa. Hence, the degree of deacetylation (DDA) has a significant influence on the thermal stability of chitosan and for more deacetylated samples, the increasing intramolecular and intermolecular hydrogen bonding helps to enhance the thermal stability [29]. In addition, lower Mw diminishes the entanglement of the polymer chains decreasing the thermal stability. Figure 2c,d also show that the grafting with DEAE and Dod groups resulted in significant changes in the TG (DTG) analysis of derivatives when compared to the starting sample CH_L_. The first thermal event occurred in the range from 30 to 200 °C and it is mainly attributed to water loss. Moreover, the water content increased with the insertion of DEAE and dodecyl groups, from 7% for CH_L_ to 8.6% and 10.1%, for DEAE-CH_L_ and DEAE-CH_L_-Dod, respectively. The insertion of functional groups can break the intramolecular and intermolecular hydrogen bonding interaction changing the relative amounts of adsorbed and bound water molecules via hydrogen bonding [29]. Besides, as a result of the reinforcement of hydrophobic interactions, the maximum degradation temperature was displaced to higher temperatures indicating an increase in thermal stability provided by the DEAE and Dod groups. Table 2 shows all these data together and reveals that, for amphiphilic derivatives, the residual mass was found to decrease with the grafting of DEAE and Dod (Figure 2c), with respective values of 27% and 20.9% for DEAE-CH_L_ and DEAE-CH_L_-Dod, respectively. 

### 2.4. Film Solubility

**Figure 3:**
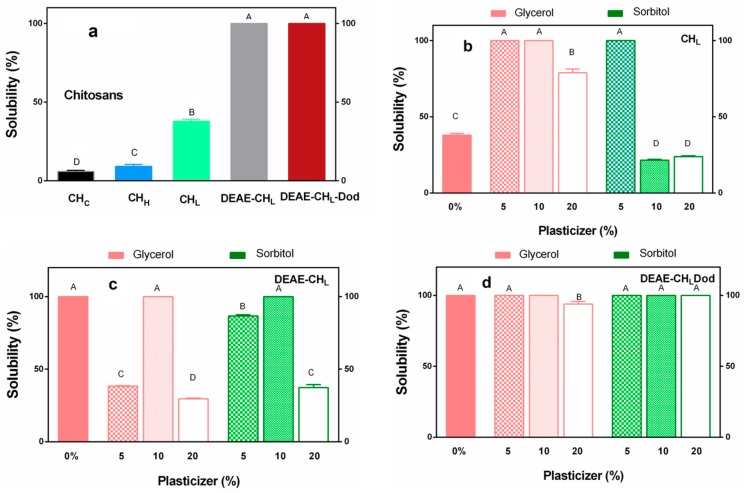
(**a**) Solubility of chitosan films without plasticizer and prepared with 5%, 10%, and 20% of glycerol and sorbitol for (**b**) CH_L_ (**c**) DEAE-CH_L_ and (**d**) DEAE-CH_L_-Dod. The values are expressed as means ± SD (*n* = 5). Different letters in the same graph indicate a significant difference (*p* < 0.05). The values are expressed as means ± SD (*n* = 3).

Film solubility is a very important property, particularly for edible films where solubility may be a required property. The study of solubility allows a more suitable choice when using the film in practice since, for certain purposes such as the protection of foods against microbial action, films of low solubility are needed while for their use as edible coating, moderate to high solubilities may be required [8,30]. Figure 3a shows the solubility in water of films without plasticizer. In general, lower Mw chitosan CH_L_ and its respective derivatives provided films with high solubility in water, reaching 100% for both DEAE-CH_L_ and DEAE-CH_L_-Dod (Figure 3a). On the other hand, CH_C_ and CH_H_ with no plasticizer provided films whose solubilities were 6.1% and 10.2%, respectively (Table S1). The high solubility for DEAE-CH_L_ and DEAE-CH_L_-Dod can be attributed to a combination of the higher pKa (~10.0) of the tertiary amino group of DEAE with the low Mw for CH_L_ (Mw 11 kDa), since its film had a solubility of about 40% in water, in contrast to the film prepared with CH_H_ (Mw 143 kDa), whose solubility was only 10.2%. Figure 3b–d show the effect of the percentage of plasticizer on the solubility of films prepared with CH_L_ and its derivatives. Plasticizer is one of the three major components of edible films and its percentage may be tailored to control the solubility, which in turn, allows the adjustment of the film properties to suit different applications, since for some foods insoluble films are needed to enhance product integrity and water resistance [10]. In this study, it became clear that the percentages of both glycerol and sorbitol affected the solubility of all chitosan films, except the amphiphilic DEAE-CH_L_-Dod, whose films were soluble independent of plasticizer concentration (5% to 20%, Figure 3d). In contrast, the solubility of the CH_L_ and DEAE-CH_L_ of films prepared with 20% of plasticizer were less soluble than the other formulations, while for CH_C_ and CH_H_, the addition of glycerol and/or sorbitol increased the solubility of the films to 15–20% (Table S2). Overall, the results showed that plasticizer percentage may be tailored to adjust solubility of the acetylated and deacetylated chitosans, independent of their Mw, while the films prepared with the amphiphilic derivative of low Mw remained soluble for all tested percentages. 

### 2.5. Mechanical Properties

**Figure 4:**
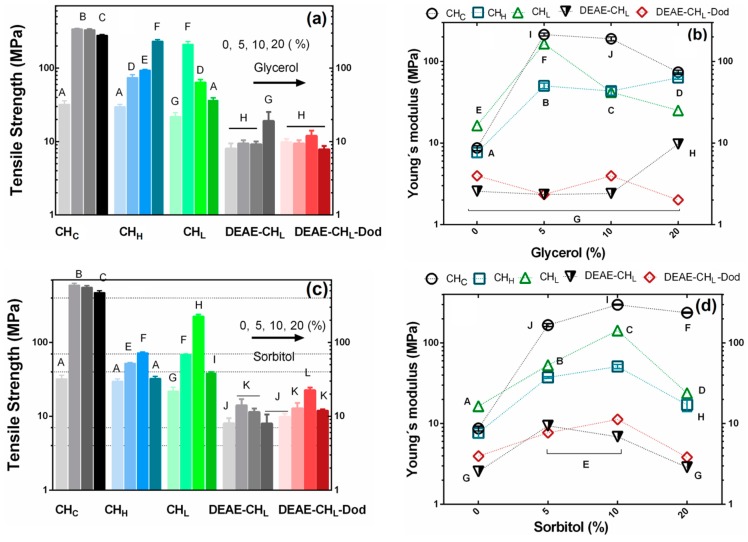
Tensile strength (TS) and Young’s modulus of chitosan films prepared with increasing concentrations of Glycerol (**a**,**b**) and Sorbitol (**c**,**d**). The values are expressed as means ± SD (*n* = 5). Different letters indicate a significant difference (*p* < 0.05).

The effect of Mw, DDA and the grafting with DEAE and Dod groups on the mechanical properties of the films was investigated by determining the tensile strength (TS, MPa), elongation at break (EB) and Young’s modulus parameters. Figure 4 shows the effects of increasing plasticizer percentages on TS and Young’s modulus. The films prepared with CH_C_, CH_H_, and CH_L_ without plasticizers had TS values of 32.06 MPa, 29.69 MPa, and 21.84 MPa, respectively (Figure 4a). These data reveal that Mw has a significant effect on TS which, as expected, increases with increasing molecular weight of chitosan [31], being similar to those recently reported for chitosans of similar Mw [29]. Moreover, glycerol (Gly) and sorbitol (Sor) similarly affected the TS and Young’s modulus, i.e., in general, the addition of Gly and Sorb at a percentage of 5% and 10% increased TS and Young’s modulus. This increase was dependent on both Mw and the chemical grafting with DEAE and Dod groups. The addition of 5% of plasticizer to CH_C_, CH_H_, and CH_L_ films increased their TS values from 2.5 to 10 times. For 20% of either Gly or Sor, the TS and Young’s modulus started to decrease, while still remaining higher than values obtained for films without plasticizers (Figure 4a,b). Moreover, it is clear that the plasticizers increased TS more abruptly for CH_C_ than they did for the films prepared with CH_H_ and CH_L_ and its derivatives, leading to values up to 590 MPa and corroborating the importance of Mw for tensile strength (Figure 4). Young’s modulus values also reflected the increasing percentages of plasticizers and the same trend was observed, making clear that the composition can be adjusted to achieve the necessary properties. 

**Figure 5:**
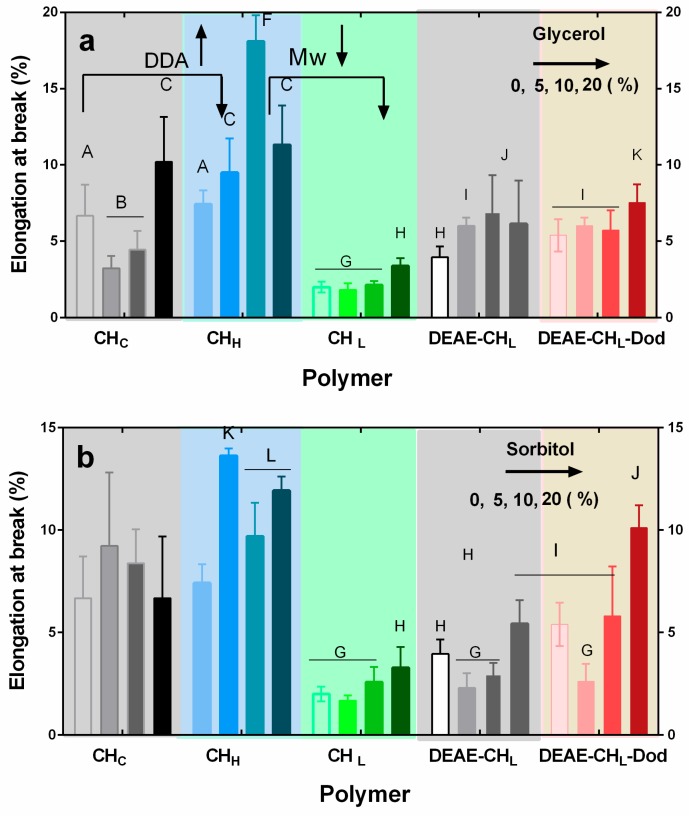
Elongation at break of films prepared with chitosan and its derivatives and increasing percentages of (**a**) glycerol and (**b**) sorbitol. The values are expressed as means ± SD (*n* = 5). Different letters indicate a significant difference (*p* < 0.05).

As shown in Figure 5, the addition of plasticizers also resulted in an increase in flexibility and extensibility, and, similar to the effects produced on TS and the Young’s modulus, the elongation at break (EB) of the films was dependent on the plasticizer structure, degree of deacetylation (DDA) and Mw. The addition of glycerol to films prepared with CH_C_ (208 kDa) and CH_H_ (143 kDa) provided the highest EB values, with maximum increases of 10.2% and 18%, respectively (Figure 5a). Moreover, the addition of glycerol resulted in slightly higher increases in EB than sorbitol, indicating that intermolecular forces between polymer coils were affected differently by the plasticizers. DDA also played an important role on EB, the deacetylation process had reduced Mw from 208 to 143 kDa, the addition of glycerol provided higher EB values for CH_H_ than for CHc. This result indicates that amino groups play an important role in providing flexibility in the films, resulting from more effective interactions between the plasticizers and the polymer chains. Moreover, increasing degrees of acetylation affect the polymer chain conformation and flexibility, and, the higher the degree of acetylation (decreasing availability of amino groups), the less flexible are the films [29]. The effect of Mw is clearly seen by comparing EB for CH_H_ (143 kDa) and CH_L_ (11 kDa), whose highest values were 18% and 3.4%, respectively (Figure 5). 

### 2.6. Water Vapor Permeability (WVP) and Thickness

**Figure 6:**
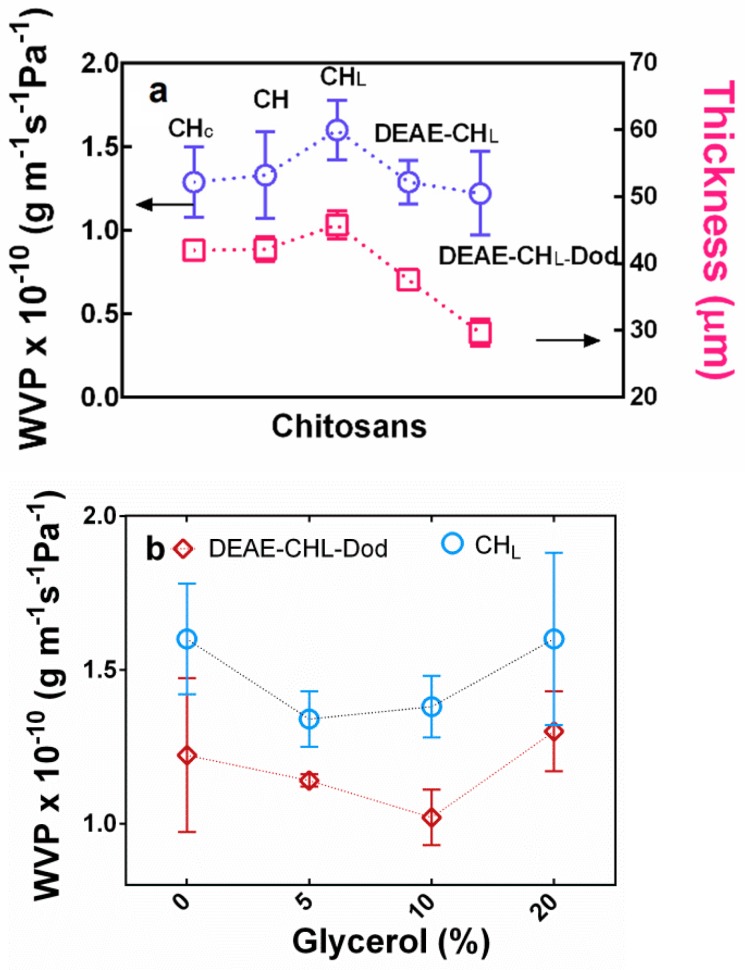

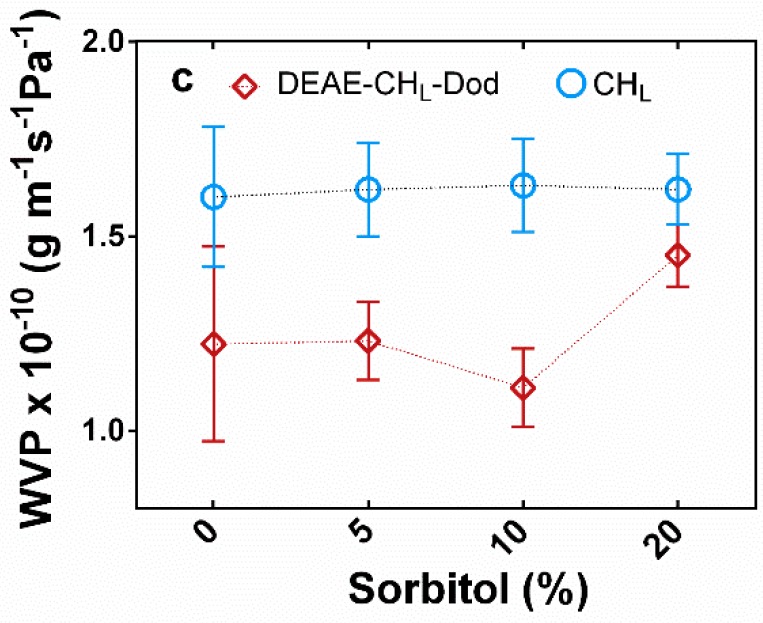
Water vapor pressure and thickness of films prepared with chitosan and its derivatives. (**a**) without plasticizer and with increasing percentages of (**b**) glycerol and (**c**) sorbitol. For statistical analysis, please see Table 3 and Table 4 (*p* < 0.05).

The water vapor permeability (WVP) is one of the most important properties due to the importance of water in deterioration of foods. It is well know that several parameters such as chemical structure, concentration, method of preparation and the presence of additives [32] may affect the water vapor permeability [33]. Hence, in this work, the sequential changes in the chitosan structure, i.e., deacetylation, degradation, and the grafting with DEAE and dodecyl groups, are expected to affect WVP since these processes will change the amphiphilic nature of the polymer chain. To evaluate the effect of structural modifications on WVP for each film, the gain in mass was determined as a function of time and the WVP rate was calculated as described in item 2.8. Figure 6a shows that WVP values for films prepared without plasticizers exhibited a tendency to increase with the deacetylation and degradation processes and then start to decrease with the insertion of DEAE and dodecyl groups. This trend can be explained by the hydrophobic nature of DEAE and dodecyl groups, which also affected the thickness of the films, decreasing them from 45 µm to 30 µm (Figure 6a) with the grafting of these groups. The higher WPV values for CH_C_, CH_H_, and CH_L_ are associated with the hydrophilic nature of chitosan provided by the protonated amino groups and their affinity to water, which is gradually decreased with the grafting by DEAE and dodecyl groups (Table 3). This trend for thickness and WVP have been also reported when oils are added to chitosan films [34] and after neutralization of the amino groups [35]. 

The addition of glycerol and sorbitol to films resulted in water vapor permeation rates ranging from 1.02 to 1.67 (10^−10^ g m^−1^ s^−1^ Pa^−1^) and these values are in close agreement with those reported by Park et al. [36]. For films prepared with CH_C_ and CH_H_, WVP only increased with sorbitol percentages. For instance, without plasticizers the WVP values for CH_C_ and CH_H_ were 1.29 and 1.33, and with the addition of sorbitol, these values increased to 1.67 and 1.65 × 10^−10^ g m^−1^ s^−1^ Pa^−1^, respectively (Table 3). On the other hand, the addition of glycerol did not significantly change the WVP for CH_C_ and CH_H_ films and, independent of the percentage of glycerol, the values remained in the range of 1.2–1.4 × 10^−10^ g m^−1^ s^−1^ Pa^−1^ (Table 3). The same trend was also observed regarding the effect of sorbitol on the thickness of CH_c_ and CH_H_ films, which increased from 42.0 and 42.2 µm to 53.1 ± 2 and 55.2 ± 1 µm, respectively, while the addition of glycerol only slightly increased the film thickness for CH_H_, from 42.2 µm to 46.8 µm. 

For the films prepared with CH_L_, DEAE-CH_L_, and DEAE-CH_L_-Dod, WVP had a tendency to decrease for formulations with 5% and 10% of plasticizer (Figure 6b and Table 3) and then changed to an increase for 20%, except for the CH_L_-sorbitol formulations which did not change and WVP remained around 1.6 × 10^−10^ g m^−1^ s^−1^ Pa^−1^ (Table 3). The addition of glycerol and sorbitol continuously increased the thickness of these films, indicating the plasticizers provide a more hydrophilic surface contributing to an increase in the adsorption of water molecules and the swelling of the films. 

### 2.7. Color Attributes and Opacity of Films

**Figure 7:**
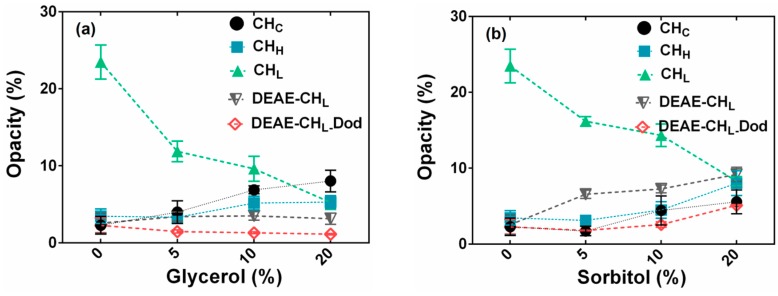
Opacity as a function of (**a**) glycerol and (**b**) sorbitol percentages (*w*/*w*) for the films prepared with chitosan and its derivatives.

The effects of the chemical modifications and plasticizer percentages on the opacity and color of the films are shown in Figure 7 and Table 5. The color of the chitosan films was affected by all modifications (deacetylation, degradation, and grafting) as well as by the plasticizer percentage. Commercial chitosan rendered films whose average clarity value (L) was 84.42 ± 1.80, while deacetylated (CH_H_) and degraded (CH_L_) chitosans presented values of 80.07 ± 0.83 and 67.65 ± 1.71, respectively, indicating lower transparency and slightly darker films (Table 5 and Table S2). The grafting with dodecyl groups (DEAE-CH-Dod) increased L again to 82.50 ± 055. 

These alterations in the color parameters of the CH_H_ and CH_L_ and DEAE-CH films compared to the CH_C_ film are linked to oxidation processes during both the deacetylation and degradation processes, resulting in high b* values, such as those obtained for CH_L_ (53.11 ± 1.86) and DEAE-CH (64.69 ± 1.95), with these two being the most yellow films without the addition of plasticizer. The CH_L_ film stood out from the others because it presented an orange-reddish hue, indicated by the highest value of a* (16.16 ± 1.98). The film for the amphiphilic derivative DEAE-CH-Dod displayed a similar coloration to that of CH_C_, while the former still exhibited a higher b* value (Table 5 and Table S2). This improvement resulted from the reductive environment provided by NaBH_4_ used in the reductive amination step with dodecyl groups [23]. 

Figure 7 shows that the film opacity was greatly changed by the degradation process, which significantly reduced the transparency of the CH_L_ film. The opacity values were also influenced by the addition of the plasticizers. In contrast to the CH_L_ film, whose opacity decreased with the percentage of the plasticizers, the CH_C_, CH_H_, and DEAE-CH_L_ opacity values increased from 2% to about 6–10%. Moreover, opacity for amphiphilic DEAE-CH_L_-Dod exhibited no significant change with added glycerol and, for DEAE-CH_L_-Dod films, only a small increase was observed for the films prepared with 20% of sorbitol (Figure 7). Hence, besides improving the mechanical properties, the addition of glycerol and sorbitol increased the opacities of all films, which may be an additional advantage taking into account that higher opacity values reduces the incidence of light in food products, preventing light-induced oxidation with loss of nutrients, discoloration and off-flavors [37]. In addition, films prepared with DEAE-CH_L_-Dod displayed improved clarity (L* = 82.50 ± 055) compared to films obtained with CH_L_ (Table 5).

### 2.8. Antimicrobial Activities and Toxicity of Chitosan and Its Derivatives

**Figure 8:**
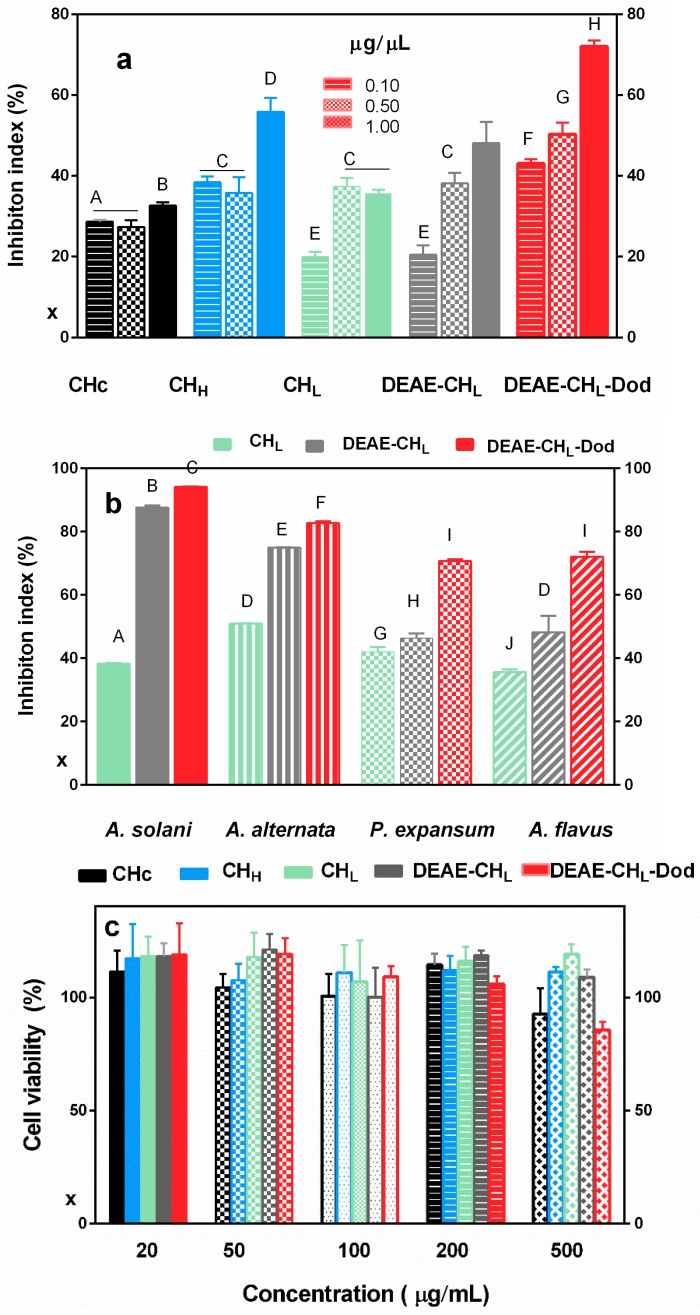
(**a**) Effect of concentration on the inhibition index of *Aspergillus flavus* and (**b**) Comparative antifungal activities of low Mw chitosans at 1.0 g/L against *Alternaria alternata*, *Alternaria solani*, *Penicillium expansum*, and *A. flavus* (**c**) NHI 3T3 cells viability as a function of polymer concentration.

The antifungal activity against *Aspergillus flavus* was evaluated from the mycelial growth as previously described [22] on the seventh day of cultivation, with the fungus being inoculated in a culture medium containing increasing concentrations of the amphiphilics. The antifungal activity of amphiphilic chitosans against *Aspergillus flavus* has been shown to depend greatly on molecular weight and degree of grafting [23]. As can be seen from Figure 8, independent of chemical structure, all chitosan samples displayed some antifungal activity against *Aspergillus flavus* and the inhibition indexes increased in a concentration-dependent manner. This activity of chitosan has been mainly attributed to the adsorption of chitosan chains on the cell wall and the cell membrane, triggered mostly by the electrostatic interactions, affecting the spores and the conidiophores population [23]. Hence the increase of DDA from 77% to 97% resulted in higher inhibition indexes for CH_H_ than for CH_C_, as a result of the increased availability of amino groups (Figure 8a and Table 1). For CH_L_, the inhibition ability decreased, which can be attributed to the lower Mw of this sample which in turn may decrease the adsorption on cell wall of the fungi. However, as seen in Figure 8a, the grafting with DEAE and dodecyl groups significantly improved (*p* < 0.05) the inhibition index, reaching more than 70% for grafted chitosan DEAE-CH_L_-Dod at 1.0 µg/µL (Figure 8a).

The filamentous fungi *A. solani*, *A. alternata,* and *P. expansum* are among the causal agents of rot in various fruits [38] being responsible for losses during storage. For example, *A. solani* is a fungal pathogen responsible for a disease in tomato called early blight. The inhibition indexes for these fungi were determined as reported earlier [39,40]. The chemical grafting also improved the antifungal activity of the starting CH_L_, and, at a concentration of 1.0 g/L, the most effective derivative DEAE-CH_L_-Dod inhibited the growth of *A. solani*, *A. alternata*, and *P. expansum*, by 94.2%, 83%, and 71%, respectively (Figure 8b). Antifungal activities of various chitosans and their derivatives against these fungi have been reported by many authors. Guo et al. [41] reported an inhibitory index of about 50% against *A. solani* by deacetylated chitosan (DDA 97%) at a concentration of 0.5 g/L, while Younes et al. [42] indicated that chitosans with lower degrees of acetylation were more efficient against *A. solani* without MW dependence. These findings confirm the importance of positive charge density for the antifungal activity of chitosan, as shown for the higher activity of CH_H_ when compared to CHc against *A. flavus* (Figure 8a). Badawy et al. have also reported the antifungal activity of N-(cinnamyl) chitosan derivatives against *Alternaria alternata*, as evaluated from in vitro mycelial radial growth technique [43]. These authors found that the inhibition of 50% of the mycelial growth (EC_50_, 672 mg/L) of *A. alternata* was more effective with the derivative N-(o-methoxycinnamyl) chitosan, suggesting that the amphipathic nature of the derivative is important for improving the antifungal activity of chitosan, as seen in the present study for DEAE-CH_L_ and DEAE-CH_L_-Dod derivatives against *A. solani* and *A. alternata* in Figure 8b. The inhibition of the mycelial growth of *P. expansum* provided by the films of CH_L_ and its derivatives displayed the same trend. The inhibition provided by CH_L_ was about 40%, similar to that obtained against the other fungi tested and in agreement with that recently reported for chitosan of high molecular weight (350 kDa) [44]. Overall, the grafting with DEAE and dodecyl groups significantly (*p* < 0.05) improved the antifungal activity of CH_L_, making these derivatives potential coatings and edible films for seeds, nuts, and fruits.

The potential application of chitosans as edible films and coating materials is based on their nontoxic nature. Chitosan itself is considered a nontoxic polysaccharide and this has been confirmed by studies using chitosan of different molecular weights and degrees of deacetylation (DD) as well as for some of its derivatives [45]. 

However, it is well known that some groups may impart some toxicity to chitosan and this subject has been appropriately reviewed by Kean and Thanou [46]. In the present study, the grafting with these groups may compromise the nontoxic character of chitosan, and taking into account the potential application of these derivatives, the in vitro cytotoxicity of chitosan and its derivatives was evaluated for fibroblasts 3T3 cells with the MTS assay. As can be seen from Figure 8c, for concentrations up to 0.5 g/L, viability was decreased only modestly by about 5–10% for DEAE-CH and DEAE-CH-Dod, and at lower polymer concentrations, no significant difference was observed between the polymers (Figure 8c). Cytotoxicity of chitosan and its derivatives has been extensively studied and, depending on the degree of substitution, cationic groups can impart toxicity to chitosan. For example, low-Mw chitosan (20 kDa) and its derivative with low degree of quaternary groups were considered nontoxic, while its highly quaternized derivatives were cytotoxic at concentrations as low as 100 ug/mL. In contrast, the modification with hydrophobic groups has been reported as decreasing the cytotoxicity of chitosan [47,48]. 

## 3. Materials and Methods

### 3.1. Materials

Commercial chitosan (CH_C_) with a degree of deacetylation (DDA) of 85% (Polymar, Fortaleza, Brazil) was deacetylated to generate a highly deacetylated sample with a DD of 97%. Sodium acetate, acetic acid, glycerol, sorbitol and sodium hydroxide were purchased from Synth (Diadema, Brazil). 2-Chloro-*N*,*N*-diethylethylamine hydrochloride (DEAE), dodecylaldehyde, deuterium chloride (35%) in deuterium oxide and deuterium oxide were purchased from Sigma-Aldrich (St Louis, MO, USA). (Sigma Aldrich), (Synth), sorbitol (Vetec, Duque de Caxias, Brazil). Potato dextrose agar (PDA) was purchased from Acumedia Manufacturers, Inc. (Lansing, MI, USA). Water was deionized using a Gehaka water purification system (São Paulo, Brazil). Spectra/Por membranes (Spectrum Chemical, New Brunswick, NJ, USA) were employed for dialysis. All solvents were of reagent grade and used as received. 

### 3.2. Synthesis of the Amphiphilic Derivatives of Diethylaminoethyl Chitosan of Low Molecular Weight (DEAE-CH-Dod)

The amphiphilic derivatives were synthesized as previously described using a highly deacetylated chitosan sample (CH_H_, DD 97%) as starting material [22]. First, CH_H_ was modified with 2-Chloro-*N*,*N*-diethylethylamine hydrochloride (DEAE) at pH 8.0 to generate a derivative with a degree of substitution of about 40% [22]. This first derivative was subsequently degraded by sodium nitrite in acetic acid solution [49,50] to obtain the DEAE-CH_L_ derivative of low Mw. Next, the sample of low Mw was recovered by lyophilization and alkylated with dodecyl aldehyde and then reduced using sodium borohydride [51] to obtain a degree of substitution of 20%.

### 3.3. Film Preparation

Film-forming solutions of chitosan and its derivatives were prepared dissolving 2.0% (*w*/*v*) of each sample in 0.5% (*v*/*v*) acetic acid aqueous solution at room temperature (25 °C) under magnetic stirring for 24 h. After the complete solubilization, the plasticizing agents, sorbitol and glycerol, were added to polymer solutions to obtain polymer/plasticizer ratios (*w*/*w*) of 5%, 10%, and 20%. Thereafter, solutions were kept under gentle stirring for 24 h and then were poured into 9 cm diameter polystyrene plates and stored in the fume hood (without exhaustion) at room temperature for 7 days to evaporate the solvent. The effects of the plasticizers on the film properties were studied by comparing the plasticizer-added films with those prepared under the same conditions without them [52]. 

### 3.4. Storage and Thickness of Films

Before being subjected to the characterization procedures, the films were stored in desiccators with relative humidity (RH) of 53% at room temperature (~25 °C) for seven days. The average film thickness was obtained based on the average of five measurements performed in random regions of the films using an MDC-25SB digital micrometer (Mitutoyo, Aurora, CO, USA).

### 3.5. Crystal Structure

The X-ray diffraction (XRD) analysis was performed according to Li [53] using an RINT 2000 X-ray diffractometer (Rigaku, Tokyo, Japan). The films were analyzed between 3° and 60° ((2θ) being the angle of diffraction) with a step size of 0.02° using a Cu-Kα, λ = 0.154 nm, radiation at 50 kV and 30 mA.

### 3.6. Color and Opacity

The color and opacity of the chitosan films and their respective derivatives were determined using a benchtop ColorFlex colorimeter (HunterLab, Reston, VA, USA) in terms of the parameters of the CIELAB colorimetric model: L*, a* and b*. L*, the clarity dimension parameter, ranging from 0 (black) to 100 (white); the parameter a * whose values change between green (−a*) and red (+a*) and parameter b* that varies from blue (−b*) to yellow (+b*). The opacity (Y) of the films was determined using the colorimeter based on equation 1, which shows a relation of the opacity of the film compared to the black pattern (Y_b_) and the white pattern (Y_w_)

(1) $$Y=\left(\frac{Y_{b}}{Y_{w}}\right)\times 100$$

To perform the color and opacity test, circular films with a diameter of 4 cm were used and the analyses were performed in triplicate, the reading being taken at four different angles: 0°, 90°, 180°, and 270°.

### 3.7. Water Solubility

The solubility of the films in water was determined following the method reported in the literature [54,55] using Equation (2),

(2) $$S=\frac{W_{0}-W_{f}}{W_{i}}\times 100$$

where W_0_ is the weight of dried film before immersion in water at 25 °C, and W_f_ is the weight of the film after 24 h of immersion under stirring (60 rpm). After drying in a TE-395 vacuum oven (Tecnal, Piracicaba, Brazil) at 60 °C and 10 kPa, the samples were weighed until constant mass.

### 3.8. Water Vapor Permeability (WVP)

The measurements of water vapor permeability (WVP) were performed gravimetrically based on the ASTM E96-95 method [56]. For the analysis, films with a mean diameter of 6.5 cm were conditioned for 7 days at a relative humidity (RH) of 53%. The film was fitted in a permeation cell partially filled with calcium chloride (2% RH) and placed in a desiccator at 25 °C containing water (100% RH). The permeation cell was weighed once per hour for 10 h, using a balance with a resolution of 0.01 mg. The slope of weight gain (Δm, in grams) versus time (Δt, in days) was obtained by linear regression. Three replicates were performed for each film formulation. The WVP was estimated using regression analysis from Equation (3) as described in the literature [57].

(3) $$WVP=\frac{\Delta m\cdot x}{A\cdot \Delta t\cdot \Delta P}$$

where x is the film thickness, A is the permeation area (0.000804 m^2^), ΔP is the difference of the water vapor partial pressure at 20 °C across the two sides of the film.

### 3.9. Mechanical Properties

Mechanical properties: tensile strength (TS), elongation-at-break (E) and the Young’s modulus were measured with a TA.XT2 texture analyzer (Stable Micro Systems, Surrey, UK) equipped with tensile test attachments following the standard method of ASTM D882-12 [58]. The initial grip separation was set at 30 mm and the crosshead speed was set at 50 mm min^−1^. Tests were replicated five times for each type of film previously conditioned for 7 days at a relative humidity (RH) of 53%. The tests were performed at 25 °C.

### 3.10. Microbiological Assays

The strains of *Alternaria alternata*, *Alternaria solani* and *Penicillium expansum* were kindly provided by the Brazilian Agricultural Research Corporation-EMBRAPA and were cultured on potato dextrose agar (PDA). The methodology employed for the bioassay with *A. alternata*, *A. solani* and *P. expansum* was that previously described by Oliveira Jr. et al. [39] and Langvad et al. [40]. The assays were performed in 96-well plates containing liquid culture medium. The biopolymeric films were cut in small pieces and added to obtain concentrations of 0.1; 0.5 and 1.0 g L^−1^. Controls were done with the culture medium plus polymer without inoculation for reference. Fungal cultures were prepared in wells of 96-well polystyrene microtiter plates, fungal growth was monitored for 7 days by ultraviolet absorbance at a 405-nm wavelength. The time of exposure and the ideal pH for the medium for *A. alternata* were chosen based on the studies by Reddy et al. [59], Liu et al. [60], and Chen et al. [44]. For *Alternaria solani*, the studies by Rodrigues et al. [61] and Coqueiro et al. [62] were used as reference, and for *Penicillium expansum*, the studies by Yu et al. [63] and Canaver et al. [64] were used. Based on the comparison of these studies, the culture medium chosen was BDA and pH 5.0.

The methodology employed for the mycelial growth inhibition assay of *A. flavus* was that previously described [22]. The inhibition indices of all polymers on the mycelial growth of *A. flavus* were determined on the seventh day of cultivation as follows:

Antifungal Index (%) = 1 − (Da/Db) × 100.

where Da is the diameter of the growth zone in the test plates and Db is the growth zone in the control plate. All data were expressed as mean ± S.E.M. Significant differences between groups were determined using two-way ANOVA with Tukey’s multiple comparison test.

### 3.11. Cytotoxicity

Cell viabilities in the presence of chitosan and its derivatives were evaluated with 3T3 fibroblast cells. Cells were cultured in Dulbecco’s modified Eagle’s medium supplemented with 10% fetal bovine serum and 1% penicillin–streptomycin and they were used in a 5–95% CO_2_-O_2_ atmosphere at 37 °C. The cells were seeded in triplicate in 96-well culture plates at a density of 1 × 10^4^ cells/mL in 200 μL of cell culture medium per well. The cells were cultured for 24 h at 37 °C. Thereafter, they were exposed to chitosan and its derivatives (20, 50, 100, 200, and 500 µg/mL) followed by an incubation period of 24 h. Cell viability was assessed with the colorimetric CellTiter96^®^ AQueous non-radioactive cell proliferation assay (Promega Corporation, Madison, WY, USA), (MTS) and phenazine methosulfate (PMS). The absorbance was measured at 570 nm with a universal ELX 808 Microplate Reader (Bio-Tek Instruments, Winooski, VT, USA).

### 3.12. Statistical Analyses

All statistical analyses on the film properties were made using the Statistica software version 7.0 (StatSoft, Tulsa, OK, USA). Analysis of variance (ANOVA) was performed for each property. Statistically significant differences were analyzed posteriori with the Tukey test. The significance level was defined as *p* ≤ 0.05, for all tests.

## 4. Conclusions

The current study investigated the physicochemical and antifungal activity of films of chitosan modified with diethylaminoethyl and dodecyl groups. The films prepared by casting with chitosan and its derivatives were prepared and characterized regarding their mechanical, barrier, color, and antifungal properties, which can be tuned by varying the percentage of added plasticizers glycerol and sorbitol. The derivatives DEAE-CH_L_ and DEAE-CH_L_-Dod combine interesting properties that resulted from the grafting with tertiary amino groups. Firstly, they provided the chitosan with a positive charge density over a broader range of pH, and allowed greater solubility, meaning good potential for application as an edible washable film. In addition, the solubility can be adjusted by the composition with the plasticizers. The presence of the DEAE and dodecyl groups decreases the water vapor permeability and thickness of the films, while providing a significant antifungal activity against all the fungi tested and, at the same time, not imparting toxicity to chitosan. The mechanical properties can be improved by adding plasticizers, and, therefore, the appropriate adjustment of the chemical composition and molecular mass may improve other properties, expanding the potential applications of these films for coatings. Overall, the grafting with DEAE and dodecyl groups provided films with improved antifungal activities against some common fungi, conferring potential to these derivatives as coatings and edible films for seeds, nuts, and fruits. 

## Figures and Tables

**Table 1:** Properties of chitosan and its amphiphilic derivatives.

Polymer	Mw (kDa)	M_n_ (kDa)	Pdi * (M_w_/M_n_)	DDA ** (%)	DS_DEAE_ + (%)	++DS_DD_ (%)
**CH_C_**	208	108	1.92	77	-	-
**CH_H_**	143	44	3.26	97	-	-
**CH_L_**	11	4.3	2.56	97	-	-
**DEAE-CH_L_**	14	5.6	2.51	53	44	-
**DEAE-CH_L_-Dod**	-	-	-	29	44	24

* Polydispersity index; ** Degree of deacetylation; + Degree of substitution by DEAE groups; and ++ Degree of substitution by dodecyl groups. Mw: molecular weight. CH_C_ = Commercial chitosan; CH_H_: Deacetylated chitosan.

**Table 2:** Maximum degradation temperature (Tmax) values and percentages of weight loss and residual mass for the polymers.

Film	T Max (°C)	Moisture (%)	Weight Loss (%)	Residual Mass (%)
**CH_C_**	281	12.5	51.3	36.2
**CH_H_**	287	13.3	52.2	34.5
**CH_L_**	265	7.0	55.4	37.6
**DEAE-CH_L_**	280	8.6	64.4	27.0
**DEAE-CH_L_-Dod**	284	10.1	69.0	20.9

**Table 3:** Average water vapor permeability of chitosan films prepared with increasing percentages of glycerol and sorbitol at a relative humidity of 53%.

	WVP × 10^−10^ (g m^−1^ s^−1^ Pa^−1^)						
Polymer	Glycerol (%)				Sorbitol (%)		
	0	5%	10%	20%	5%	10%	20%
CH_C_	1.29 ± 0.21 ^A^	1.41 ± 0.09 ^A^	1.47 ± 0.09 ^A^	1.32 ± 0.21 ^A^	1.34 ± 0.12 ^A^	1.41 ± 0.20 ^A^	1.67 ± 0.1 ^A^
CH_H_	1.33 ± 0.26 ^A^	1.31 ± 0.08 ^A^	1.34 ± 0.40 ^A^	1.22 ± 0.04 ^A^	1.30 ± 0.23 ^A^	1.56 ± 0.54 ^A^	1.65 ± 0.5 ^A^
CH_L_	1.60 ± 0.18 ^A^	1.34 ± 0.09 ^A^	1.38 ± 0.30 ^A^	1.60 ± 0.28 ^A^	1.62 ± 0.12 ^A^	1.63 ± 0.32 ^A^	1.62 ± 0.0 ^A^
DEAE-CH_L_	1.29 ± 0.13 ^A^	1.19 ± 0.15 ^A^	1.45 ± 0.26 ^A^	1.32 ± 0.14 ^A^	1.46 ± 0.15 ^A^	1.45 ± 0.12 ^A^	1.22 ± 0.1 ^A^
DEAECH_L_-Dod	1.22 ± 0.25 ^B^	1.14 ± 0.02 ^B^	1.02 ± 0.09 ^B^	1.30 ± 0.13 ^B^	1.23 ± 0.10 ^B^	1.11 ± 0.10 ^B^	1.45 ± 0.0 ^A^

Different letters in the same row for formulations of the same plasticizer indicate a significant difference (*p* < 0.05).

**Table 4:** Average thickness of chitosan films prepared with increasing percentages of glycerol and sorbitol at a relative humidity of 53%.

	Average Thickness (µm)						
Polymer	Glycerol (%)				Sorbitol (%)		
	0	5%	10%	20%	5%	10%	20%
CH_C_	42.0 ± 1 ^B^	46.1 ± 2 ^B^	43.3 ± 2 ^B^	41.1 ± 1 ^B^	43.2 ± 2 ^B^	44.1 ± 4 ^B^	53.1 ± 2 ^A^
CH_H_	42.2 ± 2 ^C^	47.5 ± 1 ^B^	46.4 ± 1 ^B,C^	46.8 ± 1 ^B,C^	49.8 ± 4 ^B^	55.8 ± 1 ^A^	55.2 ± 1 ^A^
CH_L_	45.8 ± 2 ^A^	45.0 ±1 ^A^	43.9 ± 1 ^A^	47.3 ± 1 ^A^	44.5.± 1 ^A^	44.4 ± 2 ^A^	45.4 ± 1 ^A^
DEAE-CH_L_	37.6 ± 1 ^B^	37.8 ± 1 ^B^	39.7 ± 1 ^A^	39.5± 1 ^A^	39.9± 1 ^A^	39.3 ± 1 ^A^	39.8± 3 ^A^
DEAE-CH_L_-Dod	29.7± 2 ^C^	30.5 ± 8 ^C^	33.4 ± 1 ^B^	39.7± 2 ^A^	30.4 ± 1 ^C^	32.5 ± 1 ^B^	39.5± 1 ^A^

Different letters in the same row for formulations of the same plasticizer indicate a significant difference (*p* < 0.05).

**Table 5:** Color properties of films of chitosan and its derivatives.

		Color Parameters						
Film	L* a* b*	Glycerol				Sorbitol		
		0	5%	10%	20%	5%	10%	20%
**CH_L_**		67.65 ± 1.71 ^D^	75.77 ± 0.92 ^B^	76.13 ± 1.35 ^B^	78.07 ± 1.58 ^A^	64.26 ± 0.43 ^E^	68.85 ± 0.42 ^D^	71.42 ± 0.75 ^C^
		16.16 ± 1.98 ^A^	8.30 ± 0.45 ^D^	10.75 ± 0.61 ^C^	9.56 ± 0.61 ^C^	13.60 ± 0.82 ^B^	13.45 ± 0.70 ^B^	13.04 ± 0.86 ^B^
		53.11 ± 1.86 ^C^	47.35 ± 1.62 ^E^	50.85 ± 1.72 ^D^	53.16 ± 1.65 ^C^	63.75 ± 1.47 ^B^	63.21 ± 0.92 ^B^	69.06 ± 0.94 ^A^
**DEAE-CH_L_**		66.49 ± 0.98 ^C^	64.93 ± 2.50 ^C^	65.92 ± 1.39 ^C^	56.45 ± 0.77 ^D^	66.98 ± 1.17 ^A^	66.68 ± 0.89 ^A^	68.31 ± 0.87 ^B^
		12.31 ± 0.78 ^F^	16.76 ± 0.72 ^G^	22.54 ± 1.62 ^B^	26.29 ± 0.45 ^A^	15.70 ± 0.86 ^D^	14.23 ± 0.98 ^E^	17.13 ± 0.69 ^C^
		64.69 ± 1.95 ^D^	66.34 ± 2.85 ^C^	66.19 ± 1.61 ^D^	69.79 ± 1.02 ^E^	67.34 ± 1.78 ^A^	68.77 ± 1.24 ^B^	70.39 ± 1.00 ^C^
**DEAE-CH_L_-Dod**		82.50 ± 055 ^C^	80.80 ± 0.29 ^F^	81.98 ± 1.20 ^D^	81.17 ± 0.45 ^E^	82.94 ± 0.21 ^C^	84.10 ± 0.26 ^B^	86.23 ± 0.96 ^A^
		−0.96 ± 0.31 ^A^	−0.39 ± 013 ^B^	−0.88 ± 0.43 ^C^	−0.27 ± 0.27 ^A^	−0.89 ± 0.07 ^B^	−1.21 ± 0.05 ^C^	−1.44 ± 0.04 ^D^
		30.99 ± 2.23 ^B^	33.88 ± 0.64 ^A^	35.25 ± 1.73 ^A^	34.96 ± 1.31 ^A^	27.75 ± 0.50 ^C^	26.27 ± 0.43 ^C^	17.76 ± 0.70 ^D^

Different letters in the same row for formulations of the same plasticizer indicate a significant difference (*p* < 0.05).

## Supplementary Materials

The following are available online at https://www.mdpi.com/1422-0067/20/17/4173/s1.
