# Supplementary material for: Biopolymeric Films of Amphiphilic Derivatives of Chitosan: A Physicochemical Characterization and Antifungal Study
Source: Int J Mol Sci. 2019 Aug 26;20(17):4173. doi: 10.3390/ijms20174173 (PMC6747211; doi:10.3390/ijms20174173)
Supplement: Supplementary file 1 [file ijms-20-04173-s001.pdf]

Supplementary Materials

Biopolymeric Films of Amphiphilic Derivatives of Chitosan: Physicochemical characterization and antifungal study

Anna Carolina Rodrigues Santos Alves[1], Aline Margarete Furuyama Lima[1], Marcio José Tiera[1] and Vera Aparecida de Oliveira Tiera[1*]

[1]Departamento de Química e Ciências Ambientais, Universidade Estadual Paulista (Unesp), Instituto de Biociências Letras e Ciências Exatas (Ibilce), Câmpus São José doRio Preto, R. Cristóvão Colombo, 2265, 15054-000, SP, Brazil

*Correspondence: vera.oliveira-tiera@unesp.br; Tel.: 55 17 32212358

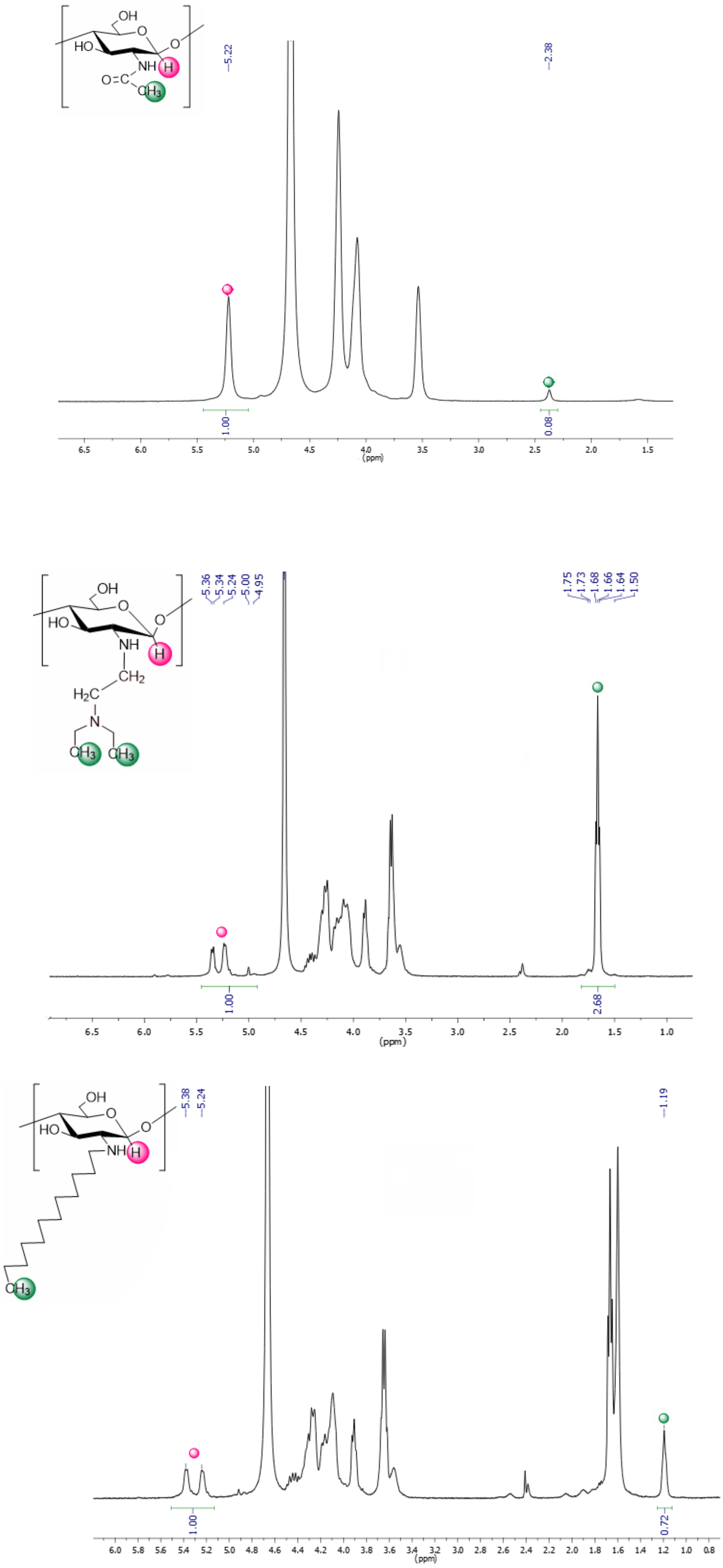

Figure S1. ^{1}H-NMR of chitosan and its amphiphilic derivatives: a) deacetylated chitosan (CHH). b) diethylaminoethyl chitosan of low molecular weight (DEAE-CHL) and the its hydrophobicized derivative.

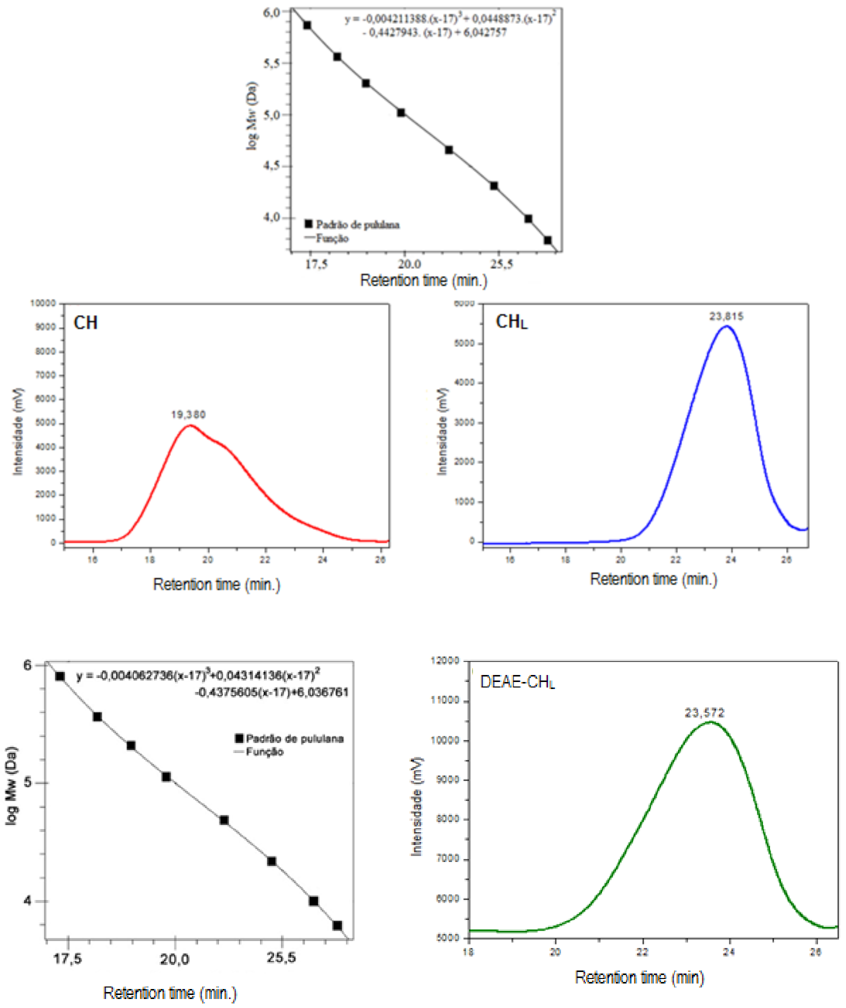

Figure S2. a) Calibration curve and chromatograms of deacetylated chitosans CH_H (143 kDa), CH_L (11 kDa) and DEAE-CH_L (14 kDa) for molecular weight determination.

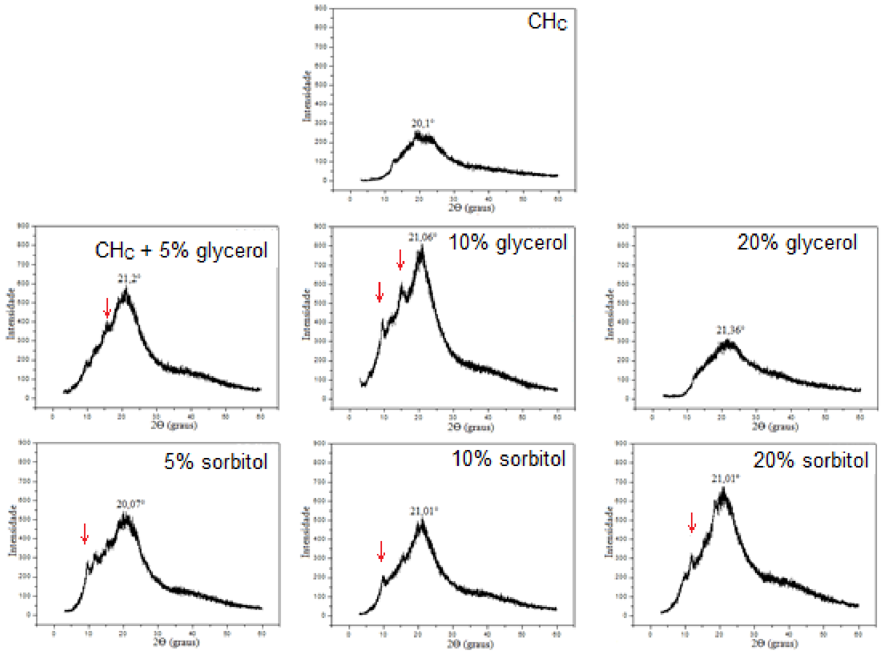

Figure S3. X-ray diffraction for films of commercial chitosan (CH_C) and its composite films containg 5%, 10% and 20% of glycerol and sorbitol. Red arrows indicate the appearance of new peaks at ~ 2θ 10-11°, not seen for CH_c without plasticizers.

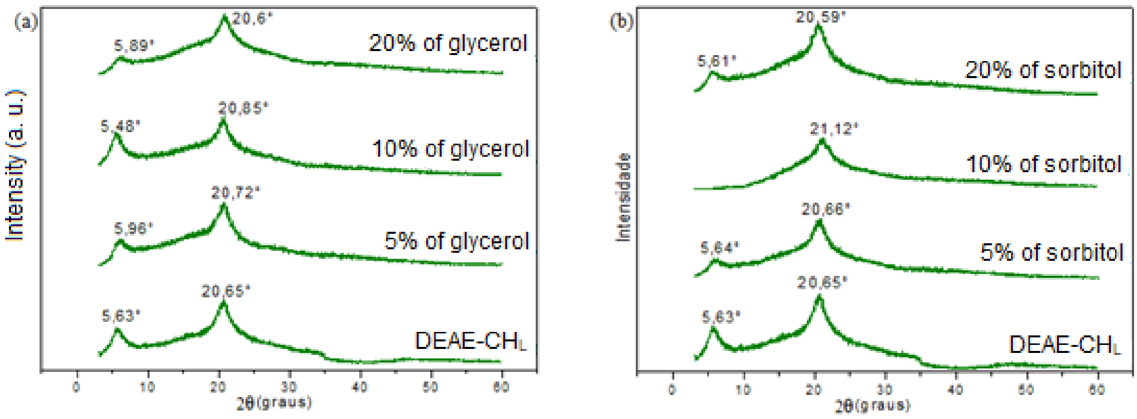

Figure S4. X-ray diffraction of diethylaminoethyl chitosan film (DEAE-CH_l) and its composite films containing 5%, 10% and 20% of glycerol and sorbitol.

Table S1– Solubility of films prepared with CH_C and CH_H at increasing concentration of glycerol and sorbitol

	Solubility of chitosan films (%)						
**Films**	**Glycerol (%)**				**Sorbitol (%)**		
	**0**	**5%**	**10%**	**20%**	**5%**	**10%**	**20%**
**CH_C**	6.10 ± 0.50	8.18 ± 0.72	8.90 ± 1.01	8.51 ± 1.17	9.48 ± 0.38	11.00 ± 0.65	15.53 ± 0.97
**CH_H**	9.22 ± 1.27	8.08 ± 0.07	10.84 ± 1.71	6.69 ± 0.28	24.24 ± 2.72	17.39 ± 2.14	21.81 ± 1.35
